# Therapeutic interventions in Osgood-Schlatter disease: A case report

**DOI:** 10.1097/MD.0000000000028257

**Published:** 2021-12-17

**Authors:** Eliza Gaweł, Anna Zwierzchowska

**Affiliations:** Institute of Sport Sciences, The Jerzy Kukuczka Academy of Physical Education in Katowice, Katowice, Poland.

**Keywords:** adolescent health, athlete, kinesiotherapy, knee injury

## Abstract

**Rationale:**

The purpose of this case study was to identify factors of bilateral etiopathogenesis of Osgood-Schlatter disease (OSD) and those supporting the effectiveness of the therapeutic process in a 12-year-old elite female Olympic karateka.

**Patient concerns:**

The present case study concerns OSD female karateka who started her sport-specific training at the age of 4 years.

**Diagnoses:**

The results of subjective palpation by the orthopedic surgeon and objective medical examination using ultrasonography, wall slide test, magnetic resonance imaging, and body height and weight measurements were collected.

**Interventions:**

The therapeutic intervention for the athlete's knee joints lasted 20 months (5 stages). Physical therapy, kinesiotherapy, and pharmacological treatment were administered, and physical activity was gradually introduced.

**Outcomes:**

The developmental trajectory was uniform for body height and labile for body weight. OSD was diagnosed after the second growth spurt, and significant progression was reported during the subsequent height and weight gains and increased volume and intensity of sports training. The rate and dynamics of changes in the distance from the patellar ligament to the tibial apophysis were irregular, with dominance in the right knee with the highest rate of change (–3.3 mm) and twice the regression of the rate of change (–2.5 mm). The analyzed distance never exceeded the baseline value (5.5 mm), which was the case in the left knee. Return to sports competition was possible from the second month of therapy, in which kinesiotherapy and static stretching were the most effective. A relatively correct distance of the patellar ligament from the tibial apophysis was recorded at the time of stabilization of the body height and weight gain. No pathological changes were observed following OSD, and full recovery was observed.

**Lessons:**

In the case discussed in this study, growth spurt, the specificity of the sport practiced, and early specialization including high-volume and high-intensity training should be considered as factors causing OSD and its progression. Kinesiotherapeutic management and static stretching are crucial for the treatment of OSD. Quick return to sports competition was possible due to early therapeutic intervention, which could also lead to the absence of pathological changes in the tibial tubercle and the absence of recurrence of OSD.

## 1 Introduction

Osgood-Schlatter disease (OSD), also known as osteochondrosis or sterile necrosis of the tibial tuberosity, is a tractional inflammatory condition of bone tissue that develops at the attachment site of the patellar tendon to the tibial tuberosity.^[1,2]^ According to Ogden et al,^[3]^ OSD is the inability of the secondary ossification center to withstand tensile forces during the growth period. OSD usually manifests itself during adolescence, mainly in boys (aged 12-15 years) participating in increased physical activity in the form of specialized sports training.^[2,4–6]^ The incidence of OSD is particularly noticeable among athletes practicing sports where movement requires jumping, running, kicking, and rapid changes in the direction of movement.^[2,4]^ Progression of the disease weakens the strength and power of the lower limbs and makes it impossible to continue the training process, which is important for the development of athletes’ sports careers. It also contributes significantly to a young person's quality of life due to pain.^[6]^

The etiopathogenesis of OSD has not yet been fully elucidated.^[1]^ The available literature indicates the most likely causes, such as excessive tension generated by the quadriceps femoris muscle, shortening of the rectus femoris muscle, inflammation of the patellar tendon and apophysis of the tibial tuberosity, and significant overload and microtrauma of the tibial tubercle.^[2,7–9]^

The structure of the tibial tuberosity was adapted to respond to the interacting traction forces. In OSD, traction forces acting on the apophysis area of the tibial tubercle lead to injury of the secondary ossification center, which consequently gradually detaches the patellar ligament from the tibial tubercle. This process results in pathological changes in the tibial tubercle.^[3]^ Further progression leads to the fragmentation of the tibial tubercle and tearing of part of the tibia with the patellar tendon (avulsion fracture), which requires surgical intervention.^[9,10]^ When a part of the bone is detached from the ossification center at the tibial tubercle, the additional formation of 1 or more bones between the detached fragments is observed.^[3]^ Most often, the disease process described in this way manifests itself in one of the limbs (boys), while bilateral cases (20%-30%) and occurrence of the disease among girls is observed less frequently.^[2,7]^ Typical symptoms of the condition include swelling of the tibial tuberosity and enlargement of the tibial tubercle. These symptoms are accompanied by pain in the subpatellar region, both in simple activities of daily living, such as climbing stairs, with an increase during physical activity.^[11,12]^ Our case study presents a unique occurrence of bilateral forms in female athletes.

## 2 Materials and methods

**Figure 1:**
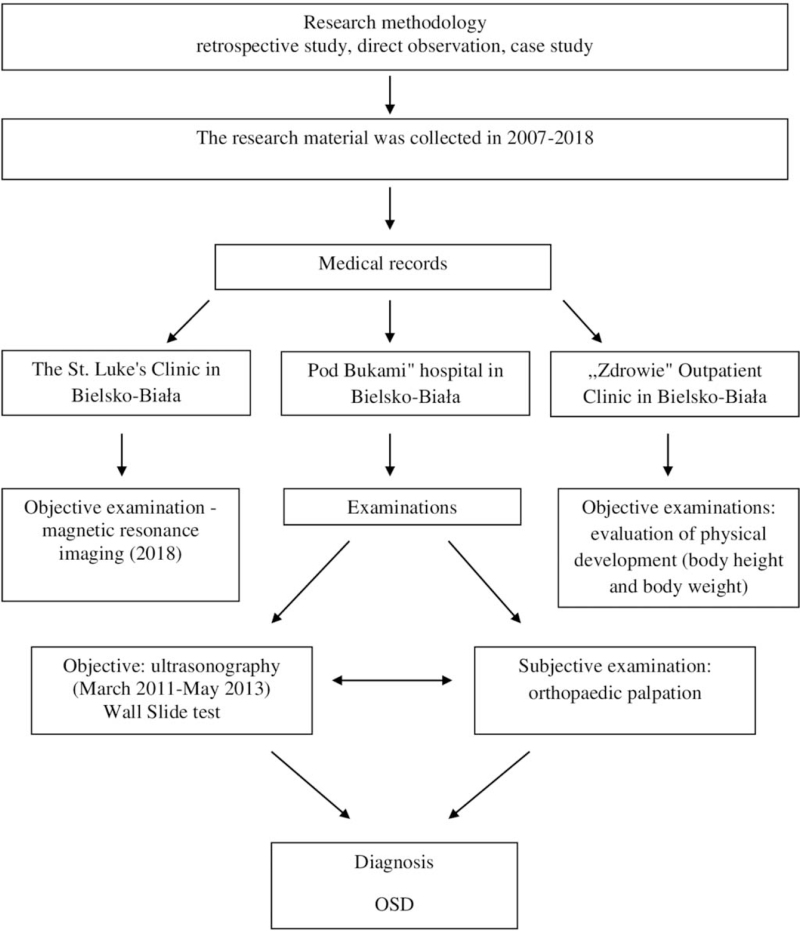
The process of documenting the research material.

Direct observation and case study methods were used in this retrospective study. The analyzed material was collected between 2007 and 2018 and consisted of subjective and objective medical examinations of a 12-year-old elite female karateka (Fig. 1). The patient provided written informed consent to use her medical data and was the subject of this case report. The study was conducted in accordance with the guidelines of the Declaration of Helsinki.

### 2.1 Case description

**Figure 2:**
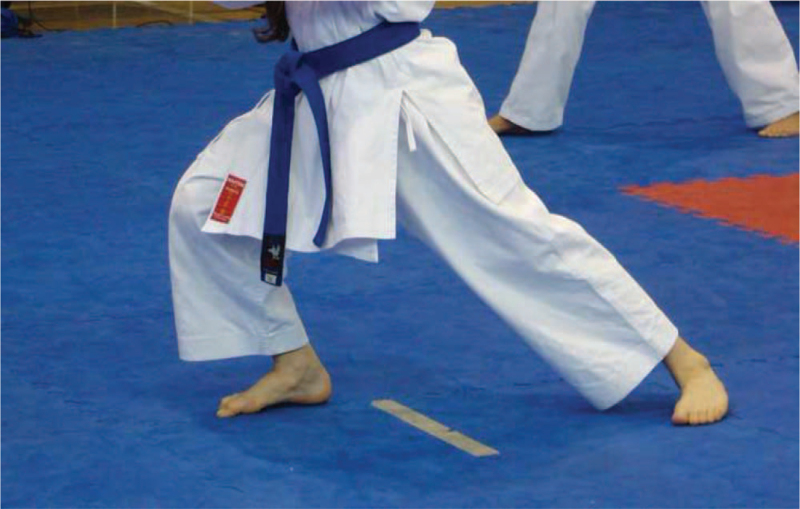
Asymmetric lower limb loading (60% on the front leg; 40% on the rear leg) with the example of the analyzed female athlete (10 yrs old).

The present case study concerns OSD female karateka who started her sport-specific training at the age of 4 years. At the time of diagnosis, her training experience was 8 years. Since she was 9 years old, the athlete has been covered by the central sports training program (competitions and training in the age group of 6 years), and she has already successfully competed in tournaments of international rank. The training load was distributed over 2 periods: the preparation period and the competitive period. The preparation period lasted 3 months, with the athlete performing 6 to 10 specialized training sessions per week at a lower intensity compared to the competitive period and with a higher volume of up to 3 hours per session. The competitive period lasted 7 months and included 6 to 8 specialized training sessions per week, with a significant volume of 2 to 3 hours per session. The training sessions took place in a gym equipped with a mat for practicing martial arts. However, for 6 years, in the first period of sports training, the athlete trained barefoot on a hard and non-cushioned surface (wood flooring). The technical specificity of the sport forces the asymmetric distribution of the athlete's body weight, with 60% resting on 1 lower limb flexed at the knee joint, and 40% resting on the other lower limb (Fig. 2).

The athlete was characterized by a very high degree of flexibility of the lower limbs and mobility of the ankle and hip joints (subjective physiotherapeutic evaluation). Furthermore, in addition to sport-specific training, the athlete practiced downhill skiing during the season of karate training (2 times a year, 14 days, 5--6 hours a day).

In 2011, a then 12-year-old elite karateka was referred to an orthopedic surgeon at the “Pod Bukami” hospital in Bielsko-Biała with a week-long persistent bilateral inflammation, swelling, and severe pain in the subpatellar region. The condition prevented her from performing athletic activities and significantly limited walking. The medical interview revealed that the progression of knee joint pain gradually increased over 18 months. Initially, the problems were labile and occurred only during or after sports training; later, they also manifested during any activity requiring knee joint flexion, for example, climbing stairs. At the same time, there was no history of knee joint injury. The orthopedist made the diagnosis by tibial palpation and ultrasonography (USG), finding bilateral advanced stage of OSD, with a prognosis of avulsion fracture and dominance of OSD in the right knee. The patient, but at the same time a karate competitor, was included in central sports training, and her physical development was monitored regularly every 6 months (March to September).

The aim of our study was to identify factors of bilateral etiopathogenesis of OSD and those responsible for the effectiveness of the therapeutic process in a retrospective study of an elite female karateka who continued to successfully practice the sport at an elite level and provided written informed consent to use her medical data and to be the subject of this case report.

### 2.2 Therapeutic intervention

**Figure 3:**
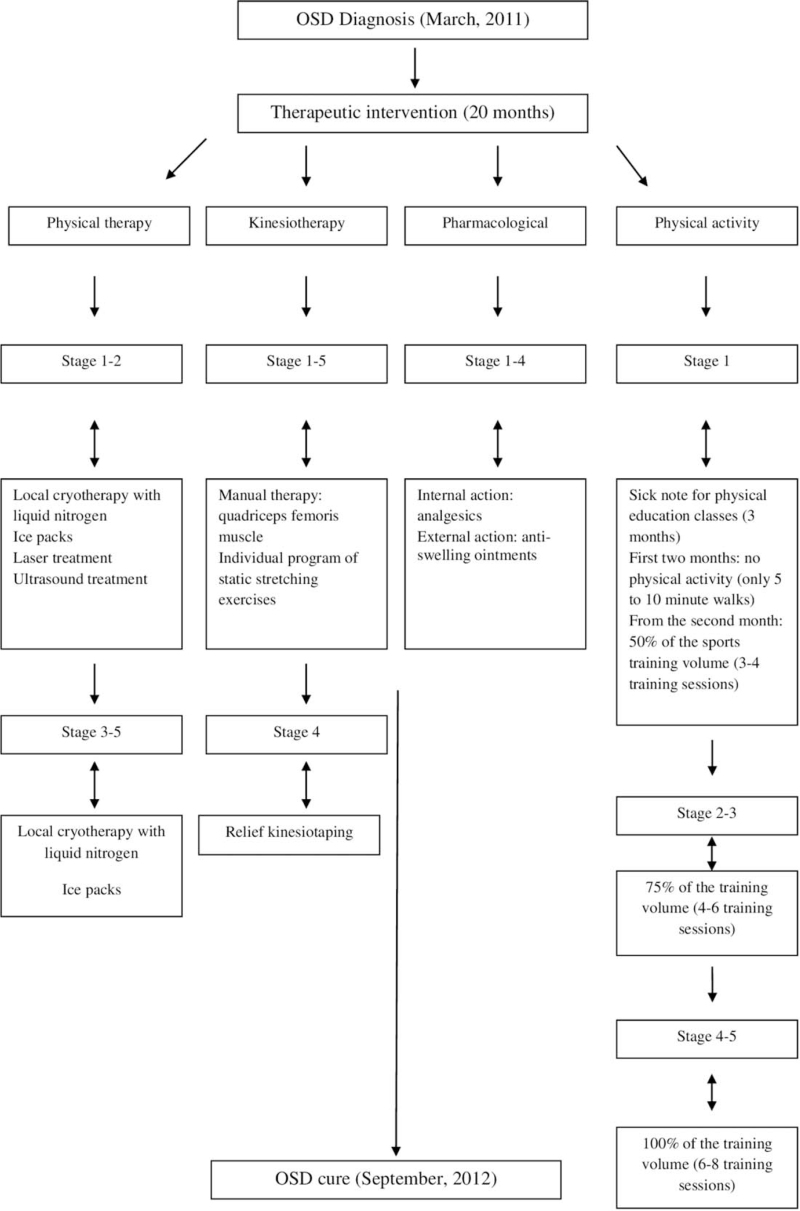
Therapeutic intervention applied to the athlete's knee joints.

The therapeutic intervention for the athlete's knee joints lasted 20 months (5 stages). Physical therapy, kinesiotherapy, and pharmacological treatment were administered, and physical activity was gradually introduced (Fig. 3).

### 2.3 Outcomes

The physical development of the studied athlete has been presented with regard to centile grids for the population of Polish girls (2007-2012).^[13]^ The developmental trajectory of body height was evenly distributed between the 90th and 97th percentiles, and the growth spurt (5 cm) was reported at the age of 11 years. In the case of body weight, its development was labile (2 centile channels). The irregularity of the physical development process and its relationship to the adolescence period was confirmed by the assessment of the body mass index (BMI) compared to the population of Polish girls (Table 1).

**Table 1:** Variability of the athlete's body height, body mass, and body mass index (BMI) compared to the population of Polish girls (2007-2012).

	Athlete's centile channel (c) compared to the population of Polish girls (2007-2012).		
Athlete's age	Athlete's body height (cm)	Athlete's body mass (kg)	Athlete's BMI (kg/m^2^)
8	141 (90-97)	34 (75-90)	17.10 (50-85)
^∗^9	144 (90-97) 146 (90-97) 151 (90-97)	37 (75-90) 40 (75-90) 41 (75-90)	17.84 (50-85) 18.76 (50-85) 17.98 (50-85)
10	154 (90-97)	43 (75-90)	18.13 (50-85)
^∗^11 (M)	157 (90-97) 162 (90-97)	47 (75-90) 51 (75-90)	19.06 (50-85) 19.43 (50-85)
12	165 (90–97) 168 (90–97)	55 (75–90) 59 (75–90)	20.20† (50–85) 20.90 (50–85)
13	170 (90-97) 172 (90-97)	59 (75-90) 64 (90-97)	20.41 (50-85) 21.60^‡^ (50-85)
14	173 (90-97) 173 (90-97)	63 (75-90) 63 (75-90)	21.04 (50-85) 21.04 (50-85)
15	173 (90-97) 173 (90-97)	63 (75-90) 70 (90-97)	21.04 (50-85)
16	175 (90-97) 175 (90-97)	72 (90-97) 70 (90-97)	
17	175 (90-97)	76 (90-97)	
18	175 (90-97)	77 (90-97)	

M = Menarche, OSD = Osgood-Schlatter disease.

∗ Athlete's growth sprut.

† OSD diagnosis.

‡ Completion of treatment for OSD.

OSD was diagnosed after the athlete's second growth spurt at the age of 11 years 10 months and 25 days and had an irregular course. In both lower limbs, after the initial regression of the patellar ligament distance from the tibial tuberosity, significant progression was reported during subsequent growth spurts in body height and body weight. During the disease, the rate and dynamics of changes were irregular, with the dominance in the right knee joint, in which the highest value of the dynamics of changes was (–3.3 mm). The rate of change was characterized by a twofold regression of (–2.5 mm). However, in the right knee, the distance of the patellar ligament from the tibial tuberosity never exceeded the baseline value (5.5 mm), which was the case in the left knee at Stages 3 and 4 (Table 2).

**Table 2:** The rate and dynamics of changes in the athlete's knee joints with respect to stages of therapy and chronological age.

		Right knee (mm)			Left knee (mm)		
OSD stages	Age chronological year of the athlete (yrs, mo, d)	Distance of the patellar ligament from the tibial tuberosity	Rate^∗^	Dynamics^∗^	Distance of the patellar ligament from the tibial tuberosity	Rate^∗^	Dynamics^∗^
1: diagnosis of OSD (March 2011)	11/10/2025	5.5	0.0	0.0	4.0	0.0	0.0
2 (July 2011)	12/02/2009	3.0	–2.5	0.0	3.0	–1.0	0.0
3 (October 2011)	12/05/2014	4.4	+1.4	–1.1	4.4	+1.4	+0.4
4 (March 2012)	12/10/2009	4.7	+0.3	–0.8	4.7	+0.3	+0.7
5 (September 2012)	13/04/2021	2.2	–2.5	3.3	2.3	–2.4	–1.7
6: orthopedic follow-up examination (07/05/2013)	14/00/2003	2.2	0.0	0.0	2.3	0.0	0.0

OSD = Osgood-Schlatter disease.

∗ Rate and dynamics of measurement of axial lateral displacement of the patella; normal mean length of the patellar ligament from the tibial tuberosity is ≤2 mm.^[14]^

The return to physical activity and sports competition was possible from the second month of therapy. The therapeutic intervention used markedly reduced the athlete's pain in the subpatellar region, which was monitored and evaluated using the Wall Slide test.

The athlete experienced the best efficiency of the therapy and pain relief after manual therapy of the quadriceps femoris muscle, and less efficiency during performing an individual set of static stretching exercises. In both cases, the results of the test indicated a progression in the depth of painless squatting compared to the test before kinesiotherapy, with higher values of deep squatting achieved in the test after the massage.

In the fourth stage of the intervention, when a significant dynamic change was observed and the pain subsided, the athlete returned to the previous volume and intensity of training. However, bilateral progression of the OSD (patellar ligament distance in the right and left knees from the tuberosity of 4.7 mm) was found shortly after starting regular training (Table 2). There was recurrent swelling of the knee joints with dominance in the right knee, which, however, did not exclude the athlete from effective training and sports competition, with ongoing therapeutic intervention.

When the patient reached a stabilized body height and weight (172 cm; 64 kg) with BMI = 21.60, a relatively normal distance from the tibial tuberosity of the patellar ligament was observed (Table 1). Orthopedic follow-up performed after 8 months confirmed the absence of OSD recurrence and ligamentous and tibial changes (Stage 6) (Table 2).

**Figure 4:**
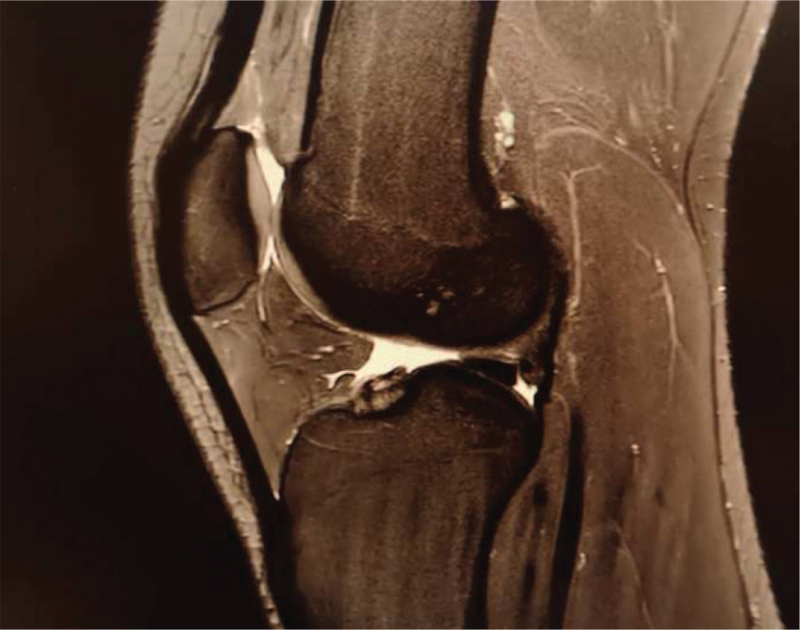
MRI image of the 19-yr-old female karateka; no pathological changes of the tibial tuberosity and ligaments are observed.

Six years later, the orthopedic follow-up was repeated using magnetic resonance imaging (MRI) (Fig. 4). No pathological changes in the tibial tuberosity were found following OSD, and full recovery was observed.

## 3 Discussion

Dynamically evolving professional sports require very early sports specialization, with athletes often coached by underqualified coaches under conditions of biomechanical overload of the musculoskeletal system, excessive loads, and negligence of general motor preparation with stretching exercises.^[7,15–17]^ This thesis is supported by the results obtained in this study, because in the analyzed case, sport-specific training (1.5-3 hours per training session) was conducted barefoot, on the floor, with little stretching and no general motor preparation. Furthermore, in the analyzed case, the training was conducted in a group with significant age differences, without taking into account the dimorphic sex-related changes between the athletes and training experience. This could have contributed to numerous micro-traumas to the athlete's musculoskeletal system, especially in the sensitive, overloaded, and underdeveloped joint structures, including the knee joints. Our findings are consistent with those presented by De Lucena et al,^[7]^ who found that training in inadequate sports facilities and lack of proper assessment of the physical condition of the young body can lead to joint damage, especially in the knees. Furthermore, reduced time of stretching exercises or failure to perform them correctly before the beginning of the sports activity may contribute to OSD. According to Gerrard,^[15]^ an immature musculoskeletal system has a lower ability to adapt to biomechanical stress. This leads to injuries in areas of increased load and dynamic musculoskeletal development, which also occurred in the present case.

Omodaka et al^[17]^ demonstrated a significant relationship between the duration of training and the onset of OSD. Hall et al^[18]^ revealed a fourfold increase in the risk of OSD when sport-specific training is performed during adolescence. Similar correlations can be indicated in the present case since sport-specific training started at the age of 4, and from the age of 6, the load and its intensity was 6 to 10 training sessions per week, which lasted up to 3 hours a day.

The diagnosis and progression of OSD in the case of the female karateka analyzed in our study occurred during the pubertal growth spurt, which is consistent with the results presented by Nakase et al,^[8]^ who noted a significant progression in the growth of the tibial tuberosity due to OSD and an increase in quadriceps femoris muscle tension while decreasing hamstring tendon tension. It was found that this phenomenon increases with age and growth processes, which leads to the presumption that the increased tension of the quadriceps femoris muscle is not only due to the change in the length of the bone (growth), but also the increased muscle strength. In the case studied, excessive repetitive tension generated by the quadriceps femoris, whose common tendon reaches the patella by attaching to the tibial tuberosity on the growth plate, can be considered the main cause of OSD. It seems that the quadriceps femoris muscle, which remains in constant excessive tension, gradually pulled up the patellar ligament, thus detaching it from its anatomical position. Undoubtedly, the training load and the specificity of the sports training (karate) with its specific posture during exercise (60% of the body weight resting alternately on 1 lower limb, bent at the knee joint) contributed to this condition. Furthermore, this process was potentiated throughout the stretch-shortening cycle of the quadriceps thigh muscle (kick, jump, and landing). Intensive loading of the knee joint during downhill skiing also causes biomechanical changes. These factors can be considered to generate not only OSD, but also its bilateral form in the analyzed case. This thesis corresponds to the findings of Itoh et al,^[19]^ who argued that movements that significantly stress the lower limb, such as kicking (in football) and 1-legged landing are associated with a higher risk of developing OSD, although these studies were conducted in male athletes.

Higher levels of muscle strength reduce the risk of injury, which has been confirmed in previous studies.^[20–22]^ Although in the analyzed case, the assessment of muscle strength was not documented, given the low body mass in relation to body height (BMI = 20.20, 50th, and 85th percentile channel; (Table 1) and lack of general motor training with an emphasis on muscle strength documented during the interview, it is reasonable to state that the strength of the lower limb muscles was low, which favored the development of OSD. This thesis is consistent with the study by Rathleff et al,^[23]^ who showed a lower level of muscle strength in the knee straightening movement in patients with OSD.

A study by Kujala et al^[24]^ found that pain associated with OSD prevented athletes from training for an average of approximately 3 months, with effective sports training possible after 7 months. In the analyzed case, the return to training and physical activity was possible after 2 months of therapy, which proves the high efficiency of the applied therapeutic intervention and appropriate adjustment of subsequent loads during sports training. Furthermore, despite the significant progression of OSD observed in the third and fourth stages, the training process could be continued after the applied therapeutic intervention in the form of massage of the quadriceps femoris muscle along with the stimulation of static stretching exercises. This led to the greatest effectiveness in regression of subpatellar pain, which was confirmed by the athlete's medical history and the wall slide test. These effects are consistent with the findings of Bezuglov et al,^[25]^ who showed that the applied therapeutic intervention (kinesiotherapeutic management) in the quadriceps femoris muscle, physiotherapy, and the gradual increase in physical activity led to discomfort during training in only 35.7% of the patients with unilateral and bilateral OSD, with the symptoms of the disease resolving spontaneously over time.

Based on their research, Ogden et al^[3]^ and Lui et al^[26]^ found that OSD usually manifests itself in adolescence and is characterized by multifactorial genesis, irregular course, and long duration of the disease, even up to several years, which was not confirmed in the present case. Circi et al^[12]^ indicated that OSD resolved spontaneously after the closure of the tibial growth plate, which is not consistent with our results because OSD did not manifest itself until after the second growth spurt, and the 20-month therapeutic and preventive intervention allowed for recovery. Furthermore, Lui et al^[26]^ found persistent OSD symptoms in adulthood, which is also not the case in the present study, and the athlete continues to practice the sport and be successful at an elite level.

The strengths of this OSD case study include the uniqueness of the occurrence of the bilateral form in the female athlete in the symmetrical sport with asymmetric loading of the lower limbs and characterized by specific movement structure (kicks, rapid changes in the direction of movements). Furthermore, the value of this study is the retrospective and direct participatory research methodology used and the description of a therapeutic intervention that was effective in the treatment of OSD, which allowed the patient to continue to practice the sport at an elite level. The collected material includes a description of the full course of the OSD, the current condition of the knee joints after OSD, and a precise profile of the physical development of the athlete (since the very beginning of sport-specific training). We also analyzed her full competitive history, which strengthened our conclusions. To our knowledge, this is the first study on OSD in female karatekas. Our case study considers the individual experience of the athlete as she is the co-author of this paper.

A limitation of the analyzed case study may be the lack of visualization of the ultrasound examination from the different stages of OSD. However, the hospital where the measurements were performed did not make its test results available in this form at that time.

## 4 Conclusions

In the analyzed case, the potential factors causing OSD and its progression include growth spurt, the specificity of the sport practiced, early sports specialization, sports training conducted in inappropriate conditions, with high volume and intensity, and poor general motor preparation with a lack of training focused on muscle strength development. Moreover, it seems that seasonal loads (alpine skiing) could have affected OSD in the present case.

The key role in the treatment of OSD in the case studied was kinesiotherapeutic management supported by manual therapy (sports massage of the quadriceps femoris muscle) and stimulation by static stretching exercises.

The athlete's quick return to sports competition was possible due to early therapeutic intervention, which may also have influenced the lack of pathological changes in the tibial tuberosity and the lack of recurrence of OSD in further stages of ontogenesis of the analyzed athlete.

## Author contributions

**Conceptualization:** Anna Zwierzchowska.

**Data curation:** Eliza Gaweł.

**Methodology:** Anna Zwierzchowska.

**Supervision:** Anna Zwierzchowska.

**Writing – original draft:** Eliza Gaweł.

**Writing – review & editing:** Eliza Gaweł.
